# Ngulluk Moort, Ngulluk Boodja, Ngulluk Wirin (our family, our country, our spirit): An Aboriginal Participatory Action Research study protocol

**DOI:** 10.1371/journal.pone.0301237

**Published:** 2024-07-03

**Authors:** Sharynne Lee Hamilton, Larissa Jones, Millie Penny, Charmaine Pell, Nicole Ilich, Carol Michie, Raewyn Mutch, Melissa O’Donnell, Carrington Shepherd, Brad Farrant

**Affiliations:** 1 Telethon Kids Institute, University of Western Australia, Perth, Australia; 2 Faculty of Health, University of Canberra, Canberra, Australia; 3 Faculty of Health Sciences, Curtin University, Perth, Australia; 4 Australian Centre for Child Protection, University of South Australia, Adelaide, Australia; 5 Ngangk Yira Institute for Change, Murdoch University, Murdoch, Australia; PLOS: Public Library of Science, UNITED KINGDOM

## Abstract

Globally, Indigenous children have historical and contemporary connections with government child protection services that have caused significant harm to their long-term health and wellbeing. Innovative, culturally secure and recovery focussed service provision is required. This paper describes a research protocol that has been designed by Indigenous researchers led by Indigenous Elders, to explore culturally secure care planning and service delivery in out-of-home care agencies in Australia. Using participatory action research methods, we will collect data using a variety of forums, including focus groups and semi-structured interviews. These data will explore the challenges for out-of-home care agencies in providing culturally secure care-planning, cultural activity and resources, and explore solutions to address factors that influence health and can assist to redress social inequities for Indigenous children. We aim to recruit approximately 100 participants for the qualitative study and 40 participants for the quantitative survey. Study participants will initially be recruited using purposive sampling, and as the study progresses will be recruited using a mixture of purposive and convenience sampling techniques. The rich data that this study is expected to yield, will inform ways to collect cultural information about Indigenous children and ways to provide cultural connections and activities that will have benefit to Indigenous children and families, and a broad range of social services.

## Introduction

Across Australia, Aboriginal and Torres Strait Islander (hereafter, respectfully termed Aboriginal) children are admitted to out-of-home care at just over 10 times the rate of their non-Indigenous peers [1] and are twice as likely to experience poor outcomes than their non-Indigenous peers, particularly in relation to mental health, and long-term wellbeing [2–7]. The inequities and subsequent poor outcomes for Aboriginal children in out-of-home care are largely attributed to a transfer of trauma across families resulting from policies of forced removal, which then contributes to the contemporary high rates of Aboriginal children removed from their families [2, 3]. Families who have been affected by these policies, commonly experience greater rates of problematic alcohol and other drug use, criminality, mental health challenges, and have low levels of social capital and support, with clinically significant behavioural problems more likely among future generations [7]. The leading causes of infant removal in Aboriginal families today includes substance misuse and maternal mental health issues, often linked to unmitigated trauma associated with repeated child removal across generations, and subsequent high levels of distrust between the Aboriginal community and state child protection services [8–11].

When children enter the out-of-home care system, their care planning is critical to ensuring their ‘best interests’ are met in out-of-home care, and they are afforded opportunities and supported to outcomes that are greater than if they had remained with their families. Consistently though, across states and territories, Australian child protection systems have failed children in out-of-home care. This is evident in the more than 50 reports and inquiries into child protection services over the last 50 years, including a Royal Commission [4] into the sexual abuse of children in institutions and foster care; an Australian Senate inquiry [5], and an extensive inquiry by the Human Rights and Equal Opportunities Commission [6] into the policies of forced removal of Aboriginal children from their kin, countries and communities.

There is global scholarship, detailing the impact on colonised Indigenous peoples and communities where dispossession and child removal have featured [12–14]. Research details poorer health and social and wellbeing outcomes for colonised Indigenous peoples and many authors have called for harm reparation [4, 6]. Addressing persistent social and health inequities requires considering both the contexts in which disparities exist, and in the case of colonised Indigenous peoples, finding innovative and culturally secure means of rectifying those inequities, which includes considering community perspectives of both the ‘causes of the causes’ and for finding solutions to address inequities [15, 16].

### Contemporary child protection landscape

In Australia, the Aboriginal and Torres Strait Islander Child Placement Principle [17] provides structured guidance for the placement of Aboriginal children removed from their families. The aim is to ensure, wherever possible, that children are placed with immediate or extended kin, or with an Aboriginal or Torres Strait Islander carer to assist to maintain connections to their kin, cultural knowledge, language and cultural activity [17–20]. Latest national figures though, show that in 2020, 57.8% of Aboriginal and Torres Strait Islander children are placed in non-Indigenous carer arrangements [1], with little tangible change to these statistics over the last five years [9]. This is of great concern. When Aboriginal children are not connected to their families and kin, and are denied opportunities for cultural immersion through activity and community interaction, there are negative consequences for their life-long health and wellbeing outcomes [18, 20].

Removing a child from their parents and family members by statutory services such as child protection authorities (often accompanied by police) is, invariably, a traumatic experience, and associated with significant losses for children. This includes a loss of personal space (family homes and bedrooms, for example), possessions, connections with broader family and friends and access to educational and social activities like school, sport or extra-curricular activity [3, 21]. Families, and the community services that advocate their needs with child protection services, including Aboriginal Community Controlled Organisations [20] can face significant relational, institutional and structural challenges to their service delivery [10, 11, 22]. Aboriginal Elders and senior leaders have consistently voiced their concern for Aboriginal children taken into out-of-home care and for the continuing erosion of knowledge of children’s connections, identity, language and cultural practices, fueling internalized racism [3, 20, 23, 24]. It is well established that Aboriginal children who know their kin, culture and community experience better health and life outcomes in the long term [20].

For Aboriginal children in out-of-home care, cultural planning is crucial for supporting and maintaining connections to kin and community, language, cultural activity and cultural knowledge. In addition to the ensuring the inclusion of children’s voices in decisions that affect them when developing cultural support plans, the amount, type and quality of information is important for them to be beneficial to children [3, 17–19]. It is also important to consider where and how cultural information is ascertained, and to ensure it is collected collaboratively with Elders and Aboriginal people specific to a child’s kin and community. In Western Australia, where this research is being conducted, the Department for Communities has responsibility for cultural planning and providing cultural connections for Aboriginal children in out-of-home care [25].

Aboriginal children are most likely to be notified to the Department for Communities for neglect and emotional abuse [25], and consistent with the national figures, around half of the Aboriginal children in care are placed with non-Indigenous carers. This is despite the calls by Aboriginal organisations and leaders for keeping Aboriginal children with their families and communities, in adherence to the Aboriginal and Torres Strait Islander Child Placement Principle [3, 19, 24]. Currently, the national policy landscape in child protection services is shifting, with a commitment to working with the national peak body for representing the interests of Aboriginal children and their families, the Secretariat of National Aboriginal and Islander Child Care, on a 10-year roadmap [25, 26]. This is designed to give greater focus to the historical and systemic issues in the child protection system, by giving an increased participatory role when decisions are made about Aboriginal children and their families [20, 26]. Regardless of these innovative policy reforms, it is likely to be some time before progress will be seen in reducing the number of Aboriginal children living in non-Indigenous care arrangements with mainstream out-of-home care agencies. This underscores the importance of working to ensure Aboriginal children in mainstream agency care are connected to their kin and culture, and that agencies have access to relevant and meaningful cultural training and support [3, 18, 20, 24].

Research has found the value and focus placed on the importance of cultural connections and cultural immersion activities for Aboriginal children is significantly different between Aboriginal and non-Indigenous agencies, including child protection workers [18, 20]. Mainstream agencies identify that there are many competing factors, such as needs that are considered a higher priority for children, that impact whether there is a greater or lesser focus on cultural connections and cultural security [20]. The Aboriginal workforce, however regardless of competing factors, places much greater value on cultural connection and cultural activity as fundamental for providing care that benefits Aboriginal children [18, 20].

The Department of Communities currently provide a commitment to developing and maintaining children’s connection with culture and kin and develop cultural support plans in collaboration with a range of internal and external Aboriginal professional and community stakeholders, including Elders and community leaders specific to a child’s community [25]. There is no known research into how child protection services conduct and manage cultural information gathering, nor how/whether this information is disseminated to relevant parties who care for children in Australia. However, Aboriginal children’s cultural plans have been found to be failing to provide cultural connections and activities [3]; and to have omissions/losses that negatively impact the long-term health and wellbeing of Aboriginal children [19, 20, 26].

A critical component for developing strong cultural identities of Aboriginal children in out-of-home care is that cultural support plans consider their place and relationships in culture and community [3, 17, 18, 24, 26]. Moreover, reunification should be prioritised in a culturally secure way [18, 20]. Given the likelihood of significant layers of intergenerational trauma for Aboriginal children, cultural plans should be flexible, well-funded and consider recovery-focused and strength-based care and should incorporate input from children in care, their parents and kinship networks [17, 18]. Strong cultural programs that support connection to traditional lands and Elders, language, kin, community inclusion, cultural activities and cultural knowledge are fundamental components for increasing resilience and wellbeing for Aboriginal children [3, 17–19].

### Applying cultural lenses

The Ngulluk Koolunga Ngulluk Koort framework, comprehensively described elsewhere [23], brings together Perth Elders’ and Aboriginal community perspectives of what is important for the development of strong Aboriginal children; the aspects of kin and community that are protective for Aboriginal children; and what Aboriginal children need for happy and healthy futures. The framework is transformational and commits to decolonising research, policy and service delivery through the provision of education and awareness about the differences in Aboriginal and other worldviews and values. Further, the way these differences are respected, to increase the capability of governments and services to develop competency for ensuring culturally secure practices are optimal when working with Aboriginal families [23]. The research identified three priority areas that are of concern to the community: (1) child protection system involvement and the impact of child removal; (2) the importance of early childhood education and care, and early schooling; and (3) housing security and homelessness for Aboriginal families.

This research protocol represents a continuation of the translation of the Elders framework and the focus on child protection/child removal research as a priority. The research draws on the community co-designed set of principles and practice recommendations for child protection work [described elsewhere: 24]. These highlight the importance of kin, culture and community and the child protection sector (government and non-government agencies) focusing on developing the Aboriginal workforce and community-controlled organisations and embracing community-identified strategies to address the social issues faced by many Aboriginal families.

The principles and practice recommendations provide practical ways of working with the Aboriginal community toward better practice in child protection service provision. The history of past child removal policies and the associated distrust of child protection services among Aboriginal communities, means that it is critically important that interventions for Aboriginal children removed from their families and placed in the care of non-Indigenous agencies and carers are co-designed by children and families and community members, are culturally secure, place-based, family-centered, and focused on building on the strengths of Aboriginal people and culture [3, 9, 18, 19, 23].

### Toward recovery

There is consensus among Aboriginal leaders and scholars that a recovery-focussed, family-centered and intergenerational approach to interventions is required to understand, and interrupt, the ongoing removal of Aboriginal children and the associated adverse health and wellbeing outcomes [3, 19]. For Aboriginal children involved with statutory authorities, community led recovery approaches have been identified as central to redressing the harm that continues to reverberate consequences across communities [27]. As it stands, the long-term health and wellbeing outcomes for Aboriginal children in foster care are poor, with youth detention and adult prisons an inevitable and at times lethal trajectory for many [28, 29]. There is a dire need for strength-based recovery-focussed policies and solutions for Aboriginal children involved with statutory systems in Australia to improve their health and wellbeing.

Recovery capital was first conceptualised as strength-based assessment tools that measure the range of internal and external resources that can be used to initiate and sustain recovery from alcohol and other drug problems and mental health issues [30]. Recovery is conceptualised at three levels: personal, social and community. Personal recovery capital represents an individual’s personal skills, abilities and personal resources including self-esteem, self-efficacy, coping mechanisms and resilience [31]. It includes individual communication skills, interpersonal and educational/vocational skills, problem-solving capacities, hope, optimism and goals. Social recovery capital refers to the recovery supports available to individuals that allow for identification of intimate relationships, family networks and broader social relationship. Community recovery capital refers to the tangible influences on recovery such as having access to safe housing, meaningful opportunities and accessible services [30, 31]. Generally, the recovery capital approach has been targeted towards adult populations. Little is known about the benefits of establishing and building on the recovery capital assets possessed by children and youth [32].

A recovery capital model constitutes a strength-based framework to assess recovery while being cognisant of trauma and subsequent mental health problems. It offers solutions and hope to the problems that are well established in children who experience child protection interventions [33]. However, the recovery capital model does not systematically consider how assessments for recovery capital assets can be applied to Indigenous children and young people living in out-of-home care, including those whose pathways find them involved with the criminal justice system. There is much potential for collecting information that allows for measuring the cultural assets of Aboriginal children, or what has been conceptualised as ‘justice capital’ [34].

### This study

The Ngulluk Moort, Ngulluk Boodja, Ngulluk Wirin (Our family, Our Country, Our Spirit) study is being conducted between 2022 and 2026 in partnership with three mainstream out-of-home care agencies and focuses on Aboriginal children living in non-Indigenous care arrangements in Perth and surrounding districts. Cultural governance and advisory groups are in place, community consultation continues to be undertaken, and initial focus groups (described below: 2.2) have been conducted. This paper describes an Aboriginal Elder-and community-led research protocol, that has been co-designed to bring together the complex dimensions associated with Aboriginal family and kinship structures, cultural connections, planning and activities. The aim is to provide out-of-home care agency workers and non-Indigenous foster carers looking after Aboriginal children, with an opportunity to have direct access to children’s Elders and Aboriginal community connections, cultural knowledge and cultural activities and resources.

The primary objectives of the research are:

1. To work with Aboriginal Elders and community, alongside stakeholders from out-of-home care agencies, to provide cultural knowledge, resources and activities to bolster current cultural support plans for Aboriginal children in their care.
2. To work with non-Indigenous foster carers and out-of-home care agency staff to develop a suite of culturally secure training and workforce support materials.
3. To provide recommendations from the research that can assist address structural challenges to collecting and sharing cultural information for Aboriginal children in care with non-Indigenous foster carers.

## Methods

This research employs a mixed methods approach utilising qualitative and quantitative methods.

### Qualitative study

#### Design

There is increasing global recognition of the imperative of including Indigenous perspectives in research. This is a response to the acknowledged historical shortcomings of research practice to protect Indigenous peoples from the continued legacies of colonisation [35, 36]. As such, using culturally secure research methods and practices is critical, to ensure research is conducted in a way that is sympathetic, respectful and ethically sound from the perspective of participants as well as prioritising Indigenous world views, wisdom, knowledge and science to inform the right way of growing up Aboriginal children [23].

**Fig 1:**
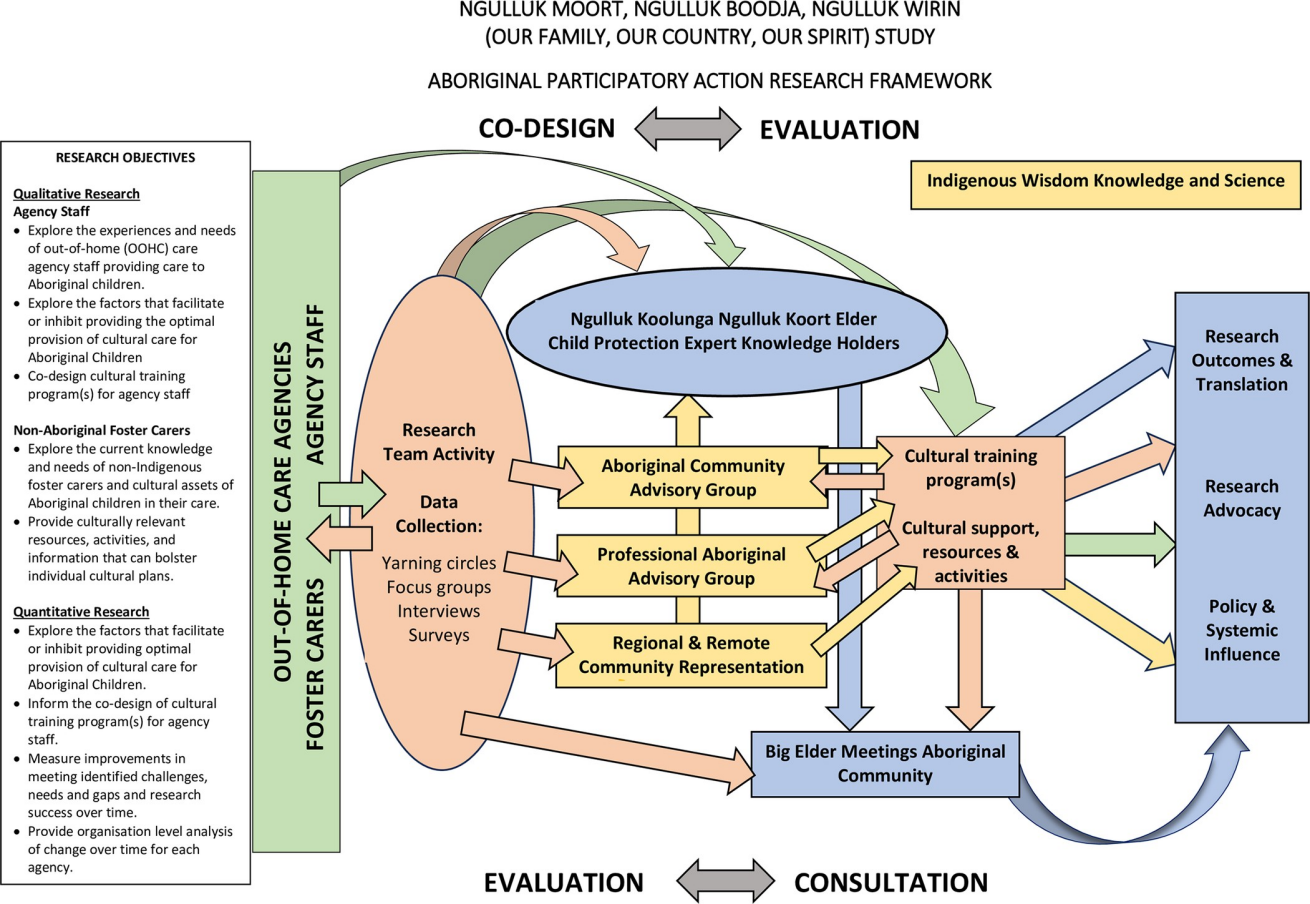
Aboriginal Participatory Action Research framework.

This research, led by the Ngulluk Koolunga Ngulluk Koort Elder child protection expert knowledge holders (hereafter, Elder expert knowledge holders), has been conceptualised and is delivered by the Elders (MP, CP), with a team of Australian Aboriginal study investigators and researchers (SLH, LJ, CM), a senior Māori academic and researcher (RM) and is supported by non-Indigenous investigators and researchers who have previously been involved in co-designing innovative, and high-quality participatory action health research (NI, MOD, CS, BF). The research places Aboriginal Elders and community at the centre by using an Aboriginal Participatory Action Research framework [36] (Fig 1) and incorporating an Aboriginal worldview and knowledge framework [36, 37]. The Aboriginal Participatory Action Research process supports a forum for combining post-colonial and hybrid knowledge in ways that inform interventions, theories, and advocacy [36]. Knowledge sharing and learning is supported through the Aboriginal Participatory Action Research process as it shifts power, shares resources, and establishes community ownership over research outcomes [36]. The Aboriginal Participatory Action Research process also supports our work and relationships with teams of non-Indigenous leaders and staff who are providing foster care services for Aboriginal children, and who provide advice and input into the policy and practice development of both their own organisations and government child protection services.

Addressing the persistent health and social inequities for Aboriginal children in out-of-home care requires consideration of the broader social and economic contexts in which disparities manifest and exploring the means to rectify those inequities [16]. Moreover, exploring innovative and culturally appropriate ways that are inclusive of community perspectives for identifying problems and solutions, or the ‘causes of the causes’ [15, 16], to address factors that influence health and social inequity. Co-designing research with and for communities using an Aboriginal Participatory Action Research approach is one way to do this. Using a strength-based co-designed approach recognises the cultural wisdom, knowledge and expertise held by the Elders, and positions ideas of family and cultural aspiration as central to the well-being of the whole community and a flourishing future. Aboriginal Participatory Action Research utilises cyclical, dynamic, and reflective processes that aid research implementation and enable community-driven solutions and is consistent with approaches that are inclusive and respectful of Aboriginal forms of wisdom and knowledge [36].

The Elder expert knowledge holders provide the cultural governance for the research. The Aboriginal Participatory Action Research design, as can be seen in Fig 1, places the community as central to all aspects of the research. We have established an Aboriginal Community Advisory Group, made up of Aboriginal community members with lived experience of out-of-home care along with a Professional Aboriginal Advisory Group comprised of Aboriginal representatives in the community sector whose work intersect with child protection services. The Elders and the research team are accountable to the broader Aboriginal community of Perth [23], and present all research activity at Big Elder Meetings, meetings which bring together a wider network of Elders for consultation, codesign and endorsement of activity. As Fig 1 shows, the Aboriginal Participatory Action Research process is an iterative, dynamic, inclusive process of co-design and consultation which encourages partnerships, and through continual evaluation and refinement, offers the best opportunity for research translation which is beneficial for participants and community [36]. The arrows show the dynamic interactions between the research objectives, the research team, the Elder expert knowledge holders, Big Elder meetings, the out-of-home care agency staff and carers, and the community. It highlights the constant, circular motion of co-design, consultation, and evaluation through to research translation.

The founding work with the agencies in this research identified that there is a significant gap in knowledge and connection with the Aboriginal community for providing optimal cultural care for Aboriginal children. As such, the development and design of this research has been informed by the following question:

Can the provision of Elder and Aboriginal community-led knowledge-sharing forums and cultural training with out-of-home care agency staff, assist to develop sustainable ways to provide cultural connections and cultural activity for Aboriginal children in the care of non-Indigenous carers?

The focus of all aspects of this research will ask questions across three key evaluation areas—research process, research outcomes, and research sustainability. First, in relation to the research process we ask: did the research work in achieving the desired aims and outcomes? Using a variety of research methods and forums we will explore whether the research is achieving the desired outcomes across its implementation, and whether it is acceptable to the Aboriginal Elders and community. In the second key area, research outcomes, we ask: was the research effective for participants? We will examine outcomes across a range of groups, including Aboriginal Elders and the community, Aboriginal children in out-of-home care, and non-Indigenous foster carers and agency staff. In the third key area, research sustainability, we ask: has this research influenced systemic change and has it provided a model for cultural competency training that could be rolled out in mainstream out-of-home care agencies?

#### Participants

The qualitative study is comprised of multiple data collection sets including out-of-home care agency staff providing case management and support to foster carers, agency non-Indigenous foster carers with Aboriginal children in their care, and participants from the Aboriginal community. Participant recruitment commenced on 16 November 2022 and will continue over the next 12 months. To date, 51 agency staff, and 27 non-Indigenous foster carers have been recruited and participated in focus groups and individual interviews. It is expected that around 100 participants will be recruited.

#### Informed consent

Fully informed written consent has been and will continue to be obtained from all study participants. Participants meet with research team members and are provided with an information statement and consent form. They are given the opportunity to ask questions about the research before providing written consent. Participation is voluntary and participants are informed that they can withdraw from the research at any point without negative consequences to their participation in any program or service. Participants are given an assurance of confidentiality in all publicly available information and peer-reviewed publications. They are advised that data and identifying information will be deidentified or coded as soon as possible, and only deidentified data will be stored on password protected computers and files that will be exclusively accessible to members of the research team.

#### Data collection

Out-of-home care agency staff will participate in several rounds of structured focus groups, led by the Elder expert knowledge holders and Aboriginal research team members. Two rounds of focus groups have been conducted. The first aimed to establish the barriers and facilitators to accessing cultural connections, knowledge, and activities for Aboriginal children in their care. The focus groups were semi-structured, exploring four themes: 1. cultural connection; 2. cultural activity; 3. cultural planning, and 4. the research partnership(s). The focus group data has provided a baseline with which to conceptualise and co-design formal and informal cultural training opportunities for the agency workers and carers.

These focus group data have been presented by the research team to the Elder expert knowledge holders, and to the cultural advisory groups and focus group participants for further data interpretation. This has informed the codesign of the necessary elements identified by the out of home care agencies for cultural training.

Focus groups will be conducted twice a year across the life of the research, for feedback, evaluation, and continual refinement of research activity. Multiple sets of semi-structured interviews will be undertaken with non-Indigenous foster carers, for the purposes of establishing the current cultural connections, resources, and activities they can provide to the children in their care, what is needed and wanted, what can be done to fill any gaps in resources and activities.

Data will be collected using forums such as yarning circles, and individual interviews employing a social yarning and research topic yarning approach [34, 38]. Yarning has become an established research method, both in Australian and global Indigenous studies, providing a safe place for Aboriginal people to share their feelings, hopes and fears through storytelling [38]. It is a fluid process of knowledge sharing and respectful communication that is flexible, allowing for adaptations that might be required to support language or literacy difference, and is suitable for both Aboriginal and non-Indigenous participants [34]. Yarning creates relationships and often reveals rich, insightful, and valuable contexts that may not be identified in traditional forms of Western interviewing [38]. Using yarning as a data collection method will allow for hearing the complexities which may exist at the intersection of the personal and community lives of participants. Aboriginal research staff will have primary responsibility for data collection to ensure that data collection activity and interactions with Aboriginal participants are culturally relevant and secure.

#### Data analysis

Qualitative analysis, data sorting, and coding will be conducted using NVivo software. A coding framework will be developed, partly from *a priori* theoretical perspective and partly from the main themes established through close reading and thematic analysis of transcripts. Multiple research team members will be involved in developing the coding frame to avoid idiosyncratic interpretation. Analysis will then be thematic, by application of this coding frame to interview transcripts and notes. The study’s Aboriginal cultural guidance groups and community members will be supported to engage with the analysis phase.

The cyclical nature of the Aboriginal Participatory Action Research (Fig 1) process includes confirming with participants the interpretations and findings of qualitative research to ensure accuracy of the views represented [36]. Analysed qualitative data will be prepared for discussion, community consultation and feedback of research findings to the Aboriginal community/s, key stakeholders, and service providers. Data will also be verified against the Consolidated Criteria for Reporting Qualitative research checklist [39].

### Quantitative study

#### Design

Informed by the intitial focus group data, a ‘Cultural Knowing, Being and Doing’ survey has been co-designed to examine organisation level comparisons over time in relation to cultural activity, cultural planning and needs. The survey will be sent to participants twice a year for three years to evaluate changes in knowledge and assess the benefits of the cultural information and activities provided over the life of the research to agency staff and carers. This offers an opportunity for continual refinement and improvement of the research activity with our partner organisations as well as providing, in the longer term, an opportunity to explore changes across the three organisations.

#### Participants

Quantitative data will be collected with around 40 agency staff who have been purposely selected through participation in the focus groups. New staff will be brought into the research as participants for both focus groups and survey participation. Agency staff were provided with an overview of the content and aims of the survey during the intitial focus groups. Information and consent to participate in the survey is required via an online consent form to proceed undertaking the survey and contact details for the ethics body and the research team are provided.

#### Data collection

SurveyMonkey will be used to develop an online, plain language survey. SurveyMonkey supports data collection through desktop and mobile applications and can be easily implemented using laptops or iPads. Where necessary the survey will be printed, and responses to hardcopy forms will be manually entered into the database.

#### Data analysis and triangulation

Quantitative data will be analysed using SPSS software Version 27. We will, primarily, undertake univariable descriptive statistics and test changes in knowledge over time, with a detailed analysis plan to be developed in collaboration with the Elder expert knowledge holders, study’s cultural advisory groups and Aboriginal community members.

Triangulation of qualitative and quantitative data will be conducted over the course of the research by the study investigators, partner organisations, members of the research team and the community. This will provide a framework for the application of interpretative lenses, which will maintain a constant culturally mediated effect on what is being heard and read and translated. The findings from the survey are expected to provide information and guidance for the agencies’ own professional cultural practices and training needs in the future.

### Ethics

This research is being conducted in accordance with and approval from the Western Australian Aboriginal Health Ethics Committee (#1137) and has reciprocal ethical approval from the University of Western Australia. Ethics amendments have been and will continue to be submitted as required for the duration of the research.

The research will be conducted in accordance with the National Health and Medical Research Council’s National Statement on Ethical Conduct in Human Research and Guidelines for Ethical Conduct in Aboriginal and Torres Strait Islander Health Research [40]. All data records will be managed according to National Health and Medical Research Council ethical guidelines and the universities standards and protocols. Audio recordings will be transferred for transcription via a share-file held on the Telethon Kids Institute’s secure drive. All data records, including audio recordings and transcripts, consent forms and survey data will be managed according to ethical requirements. Consent forms will be stored separately from any data collection forms. All hard copy and electronic data will be securely archived for a minimum of 7 years after the date of publication or research program completion, whichever is the latter, and in accordance with the requirements of the ethics bodies.

The research will honour the rights of Aboriginal peoples to have control over their cultural intellectual property, communities, resources, and Country in the creation, collection, access, analysis, interpretation, management, dissemination, and reuse of data [41].

## Evaluation translation and dissemination

This paper describes the protocol for an innovative co-designed Aboriginal Participatory Action Research [36] study, designed to mitigate the likelihood for Aboriginal children living in out-of-home care with non-Indigenous foster parents being disconnected from their cultural connections and activities [9, 17–20, 23, 24]. Furthermore, to reduce the chances of experiencing poorer life outcomes [2], mitigating the grief and loss children experience when they are removed and stemming the erosive consequences of being disconnected from family, language and cultural practices, and participating in community activities. The study is Aboriginal Elder and community-led and takes a family-centered intergenerational approach designed to understand the needs of out-of-home care agencies for providing culturally secure care for Aboriginal children living with non-Indigenous foster carers.

The unique Aboriginal Participatory Action Research [36] design shown in Fig 1 includes an iterative and constant evaluation using reflective practice. An evaluation framework for the study has been codesigned by the research team and the Elder expert knowledge holders, and this will continue to be refined throughout the research codesign process. We used a program logic approach [42] to draft the framework. Logic models identify the existing evidence that drives the intentions or aims of the research, research operations and activities allowing for accurate measurement of whether intended outcomes were achieved [42]. It is an approach which encourages stakeholders to develop a common understanding of how a program is intended to operate to achieve its objectives. In essence, a program logic approach is linear and draws a clear line from the intended aims of the research, the activities undertaken and the intended outcomes [42]. The evaluation, which will be undertaken across the life of the research through the study’s participatory action research processes, will allow us to capture unintended positive or negative consequences that result from research delivery, the general benefits or challenges for stakeholders and participants and allows flexibility to adapt the research activity as needed.

**Fig 2:**
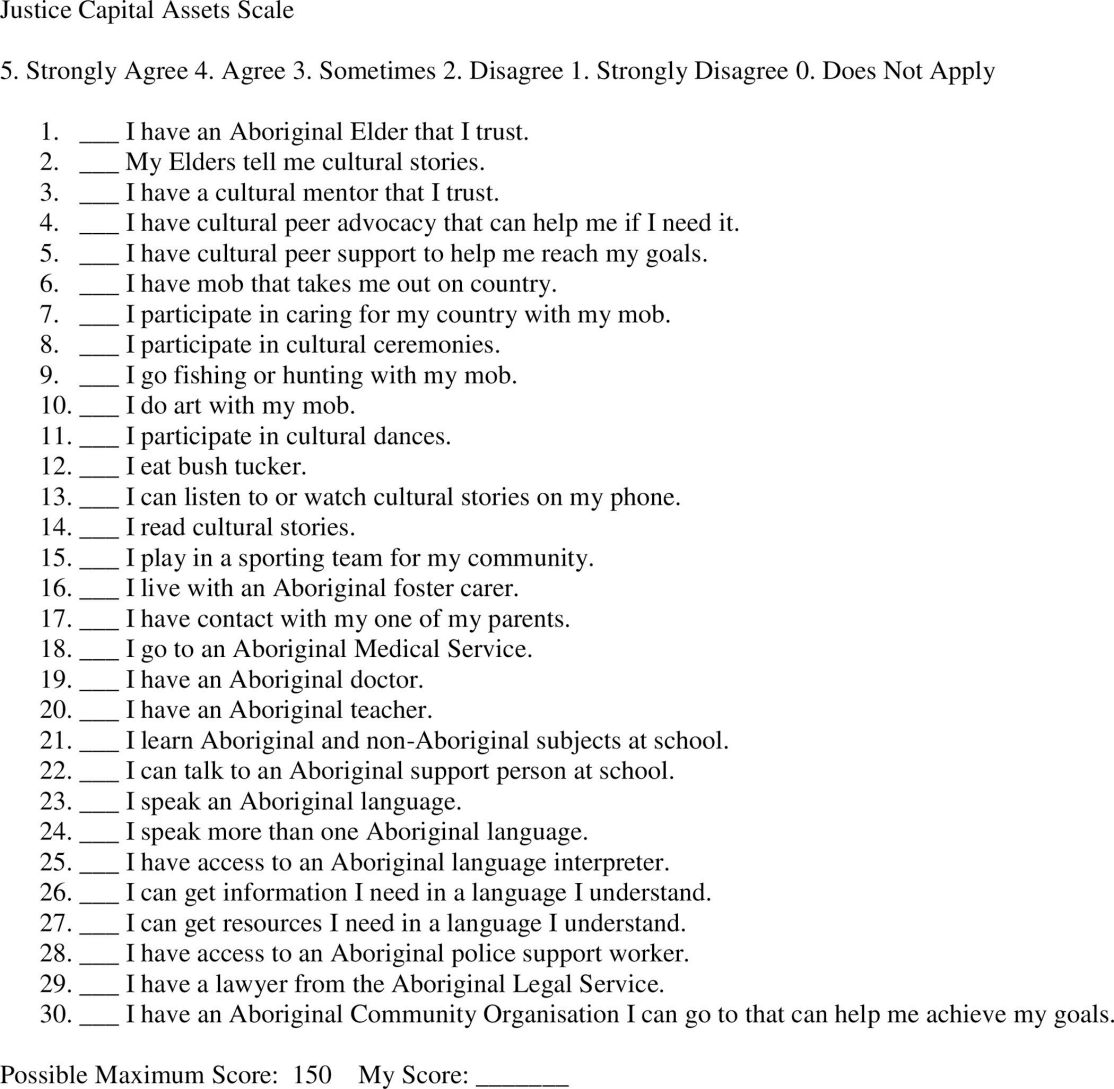
Justice capital assets scale.

Broadly, key indicators of success will be measured around Aboriginal Elder and community satisfaction, acceptability and endorsement of research outcomes and research translation. For Aboriginal children in care, success will be measured in terms of increases in cultural knowledge, connection to family and kin and opportunities for cultural activity which will be measured using a justice capital scale (Fig 2). Considering justice capital as they relate to aspects of cultural strengths and assets that are known, when absent, to contribute to child removal, and when present are protective factors for children is important. Given the likelihood of significant layers of intergenerational trauma for Aboriginal children, this measure will consider what is required to support connection to the cultural elements known to improve health and wellbeing and provide opportunities for a meaningful and culturally-connected life [17, 43–46].

The statements have been designed following extensive consultation with Elders and the Aboriginal community in Perth for both justice-involved children and those living away from home in statutory care. SLH (first author) has worked closely with Recovery Capital development experts to ensure congruence with the measures and scoring of the REC CAP [47] Scale.

For Aboriginal young people living in non-Indigenous child protection care arrangements, ensuring that all the cultural aspects that determine their health and wellbeing are considered is crucial. The higher the score, the more likely their recovery journey will provide opportunities for meaningful, happy and culturally connected lives. Lower scores provide important information about what needs to be sought to strengthen a child’s cultural connections, activity and knowledge. The justice capital scale is a self-assessment tool, and Aboriginal agency staff will support children to fill in the scale at two points in the research; prior to and after providing cultural connections, activities and resources.

For non-Indigenous foster carers, measures will be collected during qualitative interviews. We will explore foster carer views and experiences of research acceptability, engagement and inclusion. Measures will be qualitative, exploring relationships and engagement with the Elders and research team. We will explore whether carers are more connected to and engaged with the Aboriginal community and events with Aboriginal children in their care, and whether they have more knowledge about the family connections of children in their care. The ultimate measure of success will be how well the research bolsters cultural care plans that benefit children’s connections, cultural knowledge and cultural activity as identified using the justice capital assets scale (Fig 2).

Evaluation of the research for out-of-home care agency staff will explore their experiences and views about their observations of the impact of the research on children and carers as well as the benefits and challenges for foster carers and the agency staff participating in the research. In addition to the survey as an evaluation tool that allows us to refine the research activity and tailor to the needs of our partner agencies, we will use a variety of mediums such as interviews, focus groups to measure research engagement through ongoing attendance and participation. We will also explore whether there is an increase in cultural awareness and culturally secure practices through the provision of relevant and meaningful cultural training and support [48]. It is planned to develop training that both imparts knowledge through truth-telling and exploring history, exploring contemporary practice while at the same time engaging the participants in critical reflection of both their own worldviews and potential racial biases with an aim to boost cultural humility and culturally secure practice [48].

Evaluation and translation will aim to explore the factors that are required to address systemic barriers, known to impact the outcomes for Aboriginal children in care, when cultural support planning is not done well [3, 18, 19, 46]. Ultimately, the translation of this research and its effectiveness depends on how well the research relationships across the agencies, and the multiple stakeholders and participants are maintained within the Aboriginal Participatory Action Research framework [36], by understanding how well the research advocates and supports broader systemic change in the out-of-home care sector. Children could find connections with their families able to provide them kinship care, potentially increasing adherence to the Aboriginal and Torres Strait Islander Child Placement Principle [17]. There is also potential for giving an increased role in knowledge and information sharing to Aboriginal families, communities and organisations, consistent with the Department of Communities commitment to the objectives of the National Voice for our Children 10-year roadmap [26]. There is potential to provide greater knowledge and understanding about the importance placed on culture and connection for Aboriginal children in out-of-home care, bringing the differences in views between Aboriginal people and non-Indigenous agencies and child protection workers closer together [26]. The aim is for trust and confidence to be built in the Aboriginal community, and healing and reparations for past harm, by harnessing the extensive networks that exist in the Aboriginal community and promoting the potential for strong families and networks that lessen child removals for future generations and increase the chance of long-term positive social and health outcomes for Aboriginal children unable to live at home with their families.
